# Evidence That Rat Chondrocytes Can Differentiate Into Perichondrial Cells

**DOI:** 10.1002/jbm4.10056

**Published:** 2018-06-07

**Authors:** Stephan‐Stanislaw Späth, Anenisia C. Andrade, Michael Chau, Marta Baroncelli, Ola Nilsson

**Affiliations:** ^1^ Pediatric Endocrinology Unit and Center for Molecular Medicine Department of Women's and Children's Health Karolinska Institutet and University Hospital Stockholm Sweden; ^2^ Department of Medical Sciences Örebro University and Örebro University Hospital Örebro Sweden

**Keywords:** PERICHONDRIUM, GROWTH PLATE, CHONDROCYTES, DIFFERENTIATION, BONE DEVELOPMENT

## Abstract

During early bone formation, mesenchymal cells condense and then differentiate into collagen type II‐expressing chondrocytes that make up the cartilaginous bone anlagen. This anlage then becomes enclosed by the perichondrium. The mechanisms by which the perichondrium forms are not known. The purpose of this study was to determine whether epiphyseal chondrocytes can differentiate into perichondrial cells. Novel perichondrium markers were identified by expression microarray of microdissected rat perichondrium and growth plate cartilage. A dissection method that allowed for removal of contaminating perichondrium was developed and the absence was confirmed by histological examination and by expression of perichondrium markers. Perichondrium formation surrounding chondrocyte pellets was studied using histology, real‐time PCR, and in situ hybridization for chondrocyte and perichondrium markers. Cultured chondrocyte pellets developed an exterior perichondrium‐like layer. This surrounding tissue did not express chondrocyte markers, collagen‐type II and type X, as assessed by in situ hybridization. Instead, perichondrium markers, periostin, Dickkopf 3 (*Dkk3*), roundabout 2, cadherin 2, L‐galectin 1 (*Lgals1*), and thrombospondin 2 (*Thbs2*) were upregulated following formation of the perichondrium‐like layer as assessed by real‐time PCR. Interestingly, markers specific for the cambium layer, *Dkk3, Thbs2*, and *Lgals1*, but not for the fibrous layer, collagen‐type XIV and decorin, were upregulated. The findings suggest that epiphyseal chondrocytes of postnatal animals retain the potential to differentiate into perichondrial cells, supporting the hypothesis that the perichondrium originates from collagen type II‐expressing chondrocytes at the periphery of the cartilaginous bone template. © 2018 The Authors. *JBMR Plus* published by Wiley Periodicals, Inc. on behalf of American Society for Bone and Mineral Research.

## Introduction

During embryonic development, the formation of long bones starts with the condensation of mesenchymal cells that differentiate into collagen‐type II‐expressing chondrocytes.^1^, ^2^ Chondrocytes in the center of the bone template undergo hypertrophic differentiation, initiating the process of remodeling of the newly formed cartilage into bone. Simultaneously, the flanking cells form perichondrium that differentiates into periosteum and serves as a source for both trabecular and cortical osteoblasts.^2^, ^3^, ^4^, ^5^ The perichondrium consists of two layers: the inner cambium and the outer fibrous layers. The outer fibrous layer provides mechanical and structural support; the inner cambium contains progenitor cells that differentiate into osteoblasts upon Indian hedgehog (IHH) signaling from the nearby hypertrophic chondrocytes,^2^, ^6^, ^7^ and may also contribute to the appositional growth of the growth plate cartilage.^8^

The chondrogenic capacity of the perichondrium has been appreciated for some time,^9^, ^10^ whereas the capacity of chondrocytes to differentiate into perichondrium cells has only been suggested by a small number of in vitro studies.^11^, ^12^, ^13^, ^14^, ^15^ However, these previous studies did not confirm complete removal of contaminating perichondrium. It is, therefore, difficult to draw firm conclusions about the cellular origin of the perichondrium layer formed in these experiments. The hypothesis that growth plate chondrocytes have pluripotent potential which may differentiate into other skeletal cells is supported by recent lineage‐tracing experiments in mice.^5^, ^16^, ^17^

Chondrocytes cultured in monolayer gradually lose their chondrocytic phenotype and become fibroblast‐like cells.^18^, ^19^ In contrast, chondrocytes cultured in 3D pellets maintain their chondrocytic phenotype and eventually differentiate into hypertrophic chondrocytes. Chondrocyte pellet cultures also become surrounded by a perichondrium‐like layer.^13^ However, previous studies have not determined whether this perichondrium‐like layer is formed by contaminating perichondrium cells or by chondrocytes that differentiate into perichondrium cells.

To address this question, we first identified specific molecular markers of the perichondrium using expression microarray analysis of microdissected rat perichondrium and growth plate cartilage. We next developed a dissection method to isolate pieces of epiphyseal cartilage free from contaminating perichondrium. Extracted chondrocytes were then cultured in pellets; the formation of a perichondrium‐like layer was studied using histology, real‐time PCR, and in situ hybridization.

## Materials and Methods

### Animal care and handling

Sprague‐Dawley rats (Harlan, Indianapolis, IN, USA) were maintained under standard conditions and received standard rodent chow and water *ad libitum*. The protocol was approved by the Northern Stockholm Animal Ethics Committee, Stockholm, Sweden. For microdissection, 7‐day‐old male rats (*n* = 5) were killed by carbon dioxide inhalation. For the extraction of chondrocytes, epiphyseal cartilage was dissected from distal femoral and proximal tibias of 2‐ or 3‐day‐old Sprague–Dawley rats, hence before the development of a secondary center of ossification.

### Microdissection

**Figure 1:**
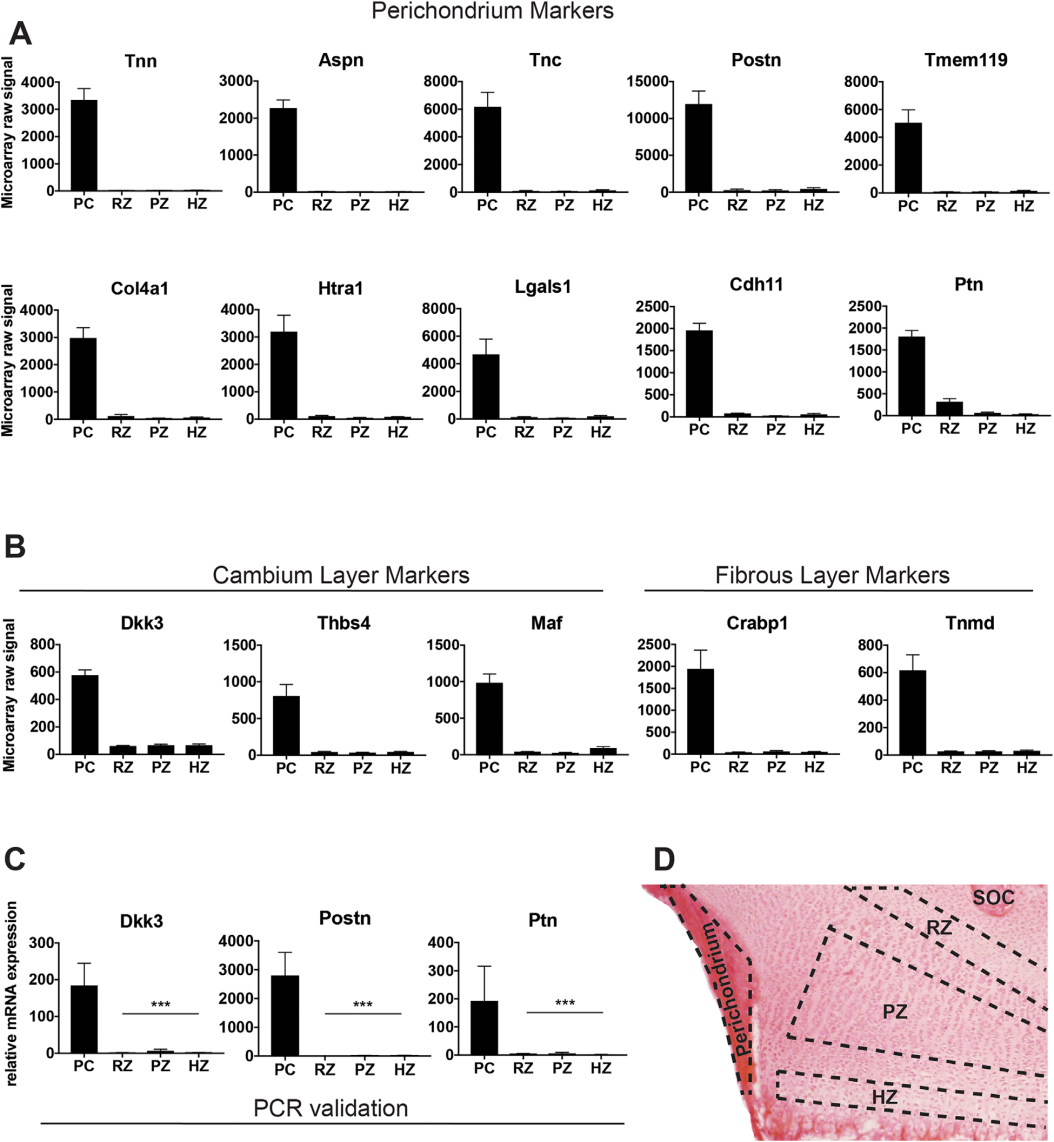
Identification and validation of perichondrium specific markers. (*A*) Microarray raw expression values of top perichondrium markers compared with individual growth plate cartilage zones. (*B*) Microarray raw expression values of selected cambium or fibrous perichondrium layer specific markers compared with individual growth plate cartilage zones. (*C*) Real‐time quantitative PCR expression data of top perichondrium markers in the perichondrium versus individual growth plate zones. (*D*) Eosin‐stained frozen sections of 1‐week‐old rat proximal tibial cartilage showing the location of perichondrium and growth plate cartilage zones that were targeted by manual microdissection. PC = perichondrium; RZ = resting zone; PZ = proliferative zone; HZ = hypertrophic zone; SOC = secondary ossification center; Tnn = tenascin N; Aspn = asporin; Tnc = tenascin C; Postn = periostin; Tmem119 = transmembrane protein 119; Col4a1= collagen type IV alpha 1; Htra1 = HtrA serine peptidase 1; Lgals1 = l‐galectin; Cdh11 = cadherin 11; Ptn = pleiotrophin; Dkk3 = Dickkopf 3; Thbs4 = thrombospondin 4; Maf = musculoaponeurotic fibrosarcoma; Crabp1 = cellular retinoic acid binding protein 1; Tnmd = tenomodulin. ***p < 0.001.

To identify perichondrium markers, mRNA expression of perichondrium was compared with individual growth plate zones using microarray analysis. Microdissection was performed as previously described.^20^ Frozen longitudinal sections (60 µm) of proximal tibial epiphyses were mounted on Superfrost Plus slides (Fisher Scientific, Chicago, IL, USA). Slides were thawed for 15 s, then placed in 70% ethanol, fixed in 100% methanol, washed in 95% ethanol, and stained in eosin (0.2% eosin, 0.5% acetic acid, 75% ethanol). Stained slides were washed in 70% ethanol, dehydrated in 100% ethanol, and then placed in xylene (each step for 1 min, at room temperature). Using an inverted microscope, razor blades, and hypodermic needles, frozen sections of proximal tibias were dissected under a xylene droplet based on histological hallmarks. First, any remaining soft tissue was cleared. The perichondrium was separated from the periosteum by a cut proximal to the hypertrophic zone (HZ)–metaphyseal bone border. Proximally, the perichondrium was separated by an oblique cut through the perichondrium at the level of the resting zone (RZ)–proliferative zone (PZ) border. The perichondrium tissue pieces were then separated from the growth plate by a gentle pull with a hypodermic needle. Only perichondrium pieces dissected clean of cartilage were included. The RZ, PZ, and HZ were then dissected as previously described.^20^ The approximate tissue pieces targeted with the microdissection are marked in Fig. 1 *D*.

### Microarray analysis

Microarray analysis was performed as described by Nilsson and colleagues.^20^ Briefly, 30 to 50 ng total RNA extracted from RZ, PZ, HZ, and perichondrium (*n* = 5 each) were processed on rat genome 230 2.0 GeneChip arrays (Affymetrix, Santa Clara, CA, USA).

Microarray signal values were quantile normalized, and the robust multiarray average background noise corrected on each array. Gene lists of spatially regulated genes were generated using Partek Genomics Suite 6.6 (Partek Inc., St. Louis, MO, USA). These expression measures were then log transformed, base 2. An ANOVA was performed for comparisons using Partek Pro software (Partek Inc.). To identify expression markers for perichondrium, we used the generated microarray data from perichondrium and individual growth plate zones. Genes highly expressed in perichondrium compared with each growth plate zone (one‐way ANOVA false discovery rate <0.01) were first selected. We then used an empirical formula (modified from^21^) that gave high rank to genes that were expressed at high levels in perichondrium and at low levels in all three zones of the growth plate (resting, proliferative, and hypertrophic): score = (R + P + H + A_PC_)/ PC, where PC = expression level in perichondrium, R = expression level in resting zone, P = expression level in proliferative zone, H = expression level in hypertrophic zone, and A_PC_ = geometric mean expression level of all genes in the perichondrium. Because an ideal marker should be expressed at a high level in the perichondrium, we included A_PC_ in the algorithm; we thus ensured that only genes abundantly expressed in the perichondrium (more than twofold higher expression level than the average gene) could receive a low score. Then genes were ranked from the lowest to the highest score. A gene was considered a potential expression marker if its expression in perichondrium and growth plate had a score of <0.5.

A heat map visualizing the difference in gene expression between the perichondrium and the growth plate of 59 top‐ranked PC markers was produced using CIMminer (https://discover.nci.nih.gov/cimminer/home.do).

### Bioinformatic analysis

Over‐represented Gene Ontology (GO) terms were analyzed by DAVID Bioinformatic Resources v6.8 (https://david.ncifcrf.gov/home.jsp) using *Rattus norvegicus* as background. Only significant enriched terms were considered (Benjamini *P *< 0.05). The genes upregulated in perichondrium were analyzed using Ingenuity Pathways Analysis software with the Disease and Function tool (IPA, QIAGEN Bioinformatics, Redwood City, CA, USA; http://www.qiagen.com/ingenuity). Pathways related to skeletal development were searched within the significantly enriched terms.

### Isolation and culture of chondrocyte pellets free of perichondrium cells

To explore the cellular origin of the perichondrium‐like layer surrounding chondrocyte pellets, we modified the dissection technique of Delgado‐Baeza and colleagues^12^ by adding a staining step that allowed for visualization and removal of all parts of the perichondrium. Briefly, distal femoral and proximal tibial epiphyses were dissected from 3‐day‐old rats removing soft tissues. Next, using a dissecting microscope the superficial layer of the articular cartilage was removed and then the epiphyseal cartilage was separated from the bone by a cut in the upper part of the hypertrophic or lower part of the proliferative zone. Each cartilage piece was then incubated in trypan blue for 1 min. This procedure caused no staining or only a faint staining of epiphyseal cartilage. In contrast, the perichondrium and other surrounding tissues were intensely stained. Next, all tissue stained blue was removed by sharp dissection under a dissecting microscope using miniature scalpels (Item# 37‐7516, Roboz Surgical Instruments Co., Gaithersburg, MD, USA) leaving a single, unstained piece of epiphyseal cartilage. This method was perfected by an investigator who consistently removed all of the perichondrium as assessed by histological analysis before the experiments were performed and then during the dissection for the definitive experiments. The cartilage pieces were pooled and 25% of the pieces were randomly selected for histological analysis; they were then fixed, paraffin embedded, cut completely into serial sections, stained with Masson trichrome, and carefully studied using microscopy to identify any remaining perichondrium. The remaining cartilage pieces were used for chondrocyte extraction as previously described with minor modifications.^22^ Briefly, cartilage pieces were washed in PBS (pH 7.4) containing 1% penicillin/streptomycin and 250 ng/mL Fungizone (Thermo Fisher Scientific, Waltham, MA, USA), incubated in shaking water bath (37°C)—first in 0.1% EDTA in PBS (15 min), then in 0.125% trypsin in PBS (30 min)—and washed twice in PBS containing 1% penicillin/streptomycin and 250 ng/mL Fungizone. Chondrocytes were extracted by digestion of cartilage pieces in 0.3% collagenase in PBS in a shaking water bath at 37°C for 4 hours. The cell suspension was filtered through a 70‐µm‐cell strainer and the chondrocytes were washed three times and then resuspended in DMEM/F12 with GlutaMAX (Thermo Fisher Scientific, Waltham, MA, USA), supplemented with 10% FBS (Thermo Fisher Scientific, Waltham, MA, USA), sodium pyruvate, penicillin (50 U/mL), streptomycin (50 µg/mL), L‐ascorbic acid (50 µg/mL), and Fungizone (0.5 µg/mL). Chondrocytes were counted using a hemacytometer and pellets were formed by aliquoting 200,000 chondrocytes in 1 mL of growth medium per 15 mL polypropylene Falcon conical tube (Becton Dickinson, Franklin Lakes, NJ, USA) followed by centrifugation at 300*g* at room temperature for 5 min. Pellet cultures were maintained at 37°C in 95% oxygen and 5% CO_2_ in a humidified chamber. The culture medium was refreshed the next day, then every other day for up to 21 days, when the cultured pellets were collected in solution C (4M guanidine thiocyanate, 25mM sodium citrate pH7, 0.1M β‐mercaptoethanol) for RNA extraction.

### Quantitative real‐time PCR

Total RNA, extracted as previously described,^23^ was reverse transcribed into cDNA and expression was quantified by real‐time PCR using an ABI Prism 7900 Fast Sequence Detector (Thermo Fisher Scientific), according to the manufacturer's instructions. Predesigned TaqMan assays (Applied Biosystems, Foster City, CA, USA) were used to assess the expression of Dickkopf 3(*Dkk3*), pleiotrophin *Ptn*, periostin (*Postn*), roundabout 2 (*Robo2*), protein tyrosine phosphatase receptor type z1 (*Ptprz1*), cadherin 2 (*Cdh2*), l‐galectin (*Lgals1*), thrombospondin 4 (*Thsb4*), collagen type XIV alpha 1 (Col14a1), collagen type II alpha 1 (Col2a1) *Col14a1*, *Col2a1*, and *18S ribosomal RNA (rRNA)*. *Col10a1* was measured by a SYBR (R) green‐based assay using custom‐designed primers designed with Primer Express 2.0 (Applied Biosystems by Thermo Fisher Scientific, Waltham, MA, USA): forward primer = GCAGCAGCCAGAATCCATTT, reverse primer = AAGTGCGCTCTTCACAACCTGT. The data were normalized to *18S rRNA* and relative expression values were calculated as previously described.^20^

### Synthesis of riboprobes for in situ hybridization

In situ hybridization was performed for perichondrium cambium layer markers *Lgals1* and cartilage markers *Col2a1* and collagen type X alpha 1 *Col10a1* in 3‐day‐old rat proximal tibias and in vitro chondrocyte pellets. DNA templates for riboprobe transcription were amplified by PCR from epiphyseal cDNA using custom‐designed primers (Table 1) containing a T7 (for sense probes) or Sp6 (for antisense probes) promoter.

**Table 1:** Designed Primers Containing a T7 (for Sense Probes) or Sp6 (for Antisense Probes) Promoter

*Lgals1*	RGD: 69355, amplicon length: 245 bp
Forward primer:	TAATACGACTCACTATAGGGAG**TCCCTTTCCAGCCTGGG**
Reverse primer:	TGGATTTAGGTGACACTATAGAAG**TGGCTTCACTCAAAGGCCAC**

*Col2a1*	RGD: 2375, amplicon length: 397 bp
Forward primer:	TAATACGACTCACTATAGGGAG**CTTGAGACAGCATGACGTCGA**
Reverse primer:	TGGATTTAGGTGACACTATAGAAG**CCGTCGCCGTAGCTGAAGT**

*Col10a1*	RGD: 2371, amplicon length: 448 bp
Forward primer:	TAATACGACTCACTATAGGGAG**AAGAGATTTCAGTAAGAGGAGAACAAGG**
Reverse primer:	TGGATTTAGGTGACACTATAGAAG**TCTGTCCATTCACACCAGGAG**

PCR products were purified by agarose gel electrophoresis and QIAquick Gel Extraction Kit (QIAGEN, Hilden, Germany). Single‐stranded riboprobes for in situ hybridization were transcribed using DIG Labeling Kit (Roche Diagnostics, Mannheim, Germany) that incorporates a digoxigenin‐ (DIG‐) conjugated uracil every 20 to 25 nucleotides. Labeled RNA probes were purified using Micro Bio‐Spin 30 Columns (Bio‐Rad, Hercules, CA, USA) and quantified using a NanoDrop Spectrophotometer (Thermo Fisher Scientific).

### In situ hybridization

In situ hybridization was performed as previously described with minor modifications.^24^ Briefly, 5‐µm‐thick sections of paraffin embedded chondrocyte pellets and decalcified bone tissue was sectioned onto Superfrost Plus slides (Thermo Fisher Scientific). Tissue sections were baked at 65°C for 1 hour, deparaffinized in xylene, rehydrated in graded ethanol baths (100%, 95%, and 70%), rinsed in DEPC‐treated water and incubated with proteinase K for 30 min (100 or 300 µg/mL in PBS, pH7.4), fixed (10% formalin, 5 min), and acetylated (0.25% acetic acid, 15 min), followed by two 5‐min PBS washes. Prehybridization was performed at 65°C for 2 hours in hybridization solution (50% formamide, 10mM Tris pH7.6, 200 µg/mL Torula yeast RNA, 1× Denhardt's Solution, 10% dextran sulfate, 600mM NaCl, 0.25% SDS, 1mM EDTA pH8.0). Hybridization with DIG‐labeled riboprobes (100 to 200 ng in 100 µL hybridization solution) was performed at 65°C for 16 hours. Posthybridization was carried out by rinsing in 4× SSC at 65°C, washing with 1× SSC and 50% formamide at 65°C for 30 min, subjecting to RNAse A digestion (10 µg/mL RNase A in RNase buffer solution—1M NaCl, 10mM Tris HCl, 1mM EDTA, pH8) at 37°C for 30 min, and washing at increasing stringency (4× SSC at room temperature for 5 min, 1× SSC at room temperature for 5 min, 0.5× SSC at room temperature for 5 min, 0.2× SSC at 55°C for 15 min). For the detection of bound riboprobes, tissue sections were rinsed in Tris wash solution (0.1M Tris, 0.15M NaCl, 0.3% v/v Tween‐20, pH7.5), blocked with 1% BSA in Tris buffer solution (0.1M Tris, 0.15M NaCl, pH7.5) for 30 min, incubated with alkaline phosphatase‐conjugated anti‐DIG antibody (Roche Diagnostics) in 1% BSA in Tris buffer solution at room temperature for 2 hours, and incubated with nitro blue tetrazolium chloride/5‐bromo‐4‐chloro‐3‐indolyl phosphate substrates (Sigma‐Aldrich, St. Louis, MO, USA) in NTM buffer solution (100mM NaCl, 100mM Tris pH9.5, 50mM MgCl_2_) at room temperature in the dark for up to 1 day until a colorimetric change was detected. For mounting, tissue sections were rinsed in PBS for 5 min, fixed in 10% formalin for 20 min, dehydrated in an ethanol series (70%, 95%, 100%) and xylene, and mounted using Permount (Thermo Fisher Scientific, Waltham, MA, USA).

### Identification of perichondrium markers

To identify expression markers for perichondrium, we selected genes highly expressed in perichondrium compared with each growth plate zone (one‐way ANOVA false discovery rate <0.01). We then used an empirical formula that gave high rank to genes that were expressed at high levels in perichondrium and at low levels in all three zones of the growth plate (resting, proliferative, and hypertrophic): score = (R + P + H + A_PC_)/ PC, where PC = expression level in perichondrium, R = expression level in resting zone, P = expression level in proliferative zone, H = expression level in hypertrophic zone, and A_PC_ = geometric mean expression of all genes in the perichondrium. Then genes were ranked from the lowest to the highest score. A gene was considered a potential expression marker if its expression in perichondrium and growth plate had a score of <0.5.

### Statistical analysis

Data are presented as mean ± SEM. Perichondrium and individual growth plate zones were collected from the same animals; therefore, each sample is not independent (several samples from the same animal). Comparison of mRNA expression levels between distinct zones of growth plate and perichondrium were performed on log‐transformed relative expression data using paired *t* tests and corrected for multiple comparisons using the Holm–Sidak method. SigmaStat 4.0 statistical program (Systat Software, San Jose, CA, USA) was used to perform all statistical measures. All *p* values were two‐tailed; significance was recognized at *p *< 0.05.

## Results

### Identification of perichondrium markers

To identify perichondrium markers that can be used to study differentiation of perichondrium cells, we developed an algorithm that ranks the genes based on their differential expression in perichondrium compared with growth plate cartilage and also prioritizes highly expressed genes as described above. Using an algorithm cut‐off of <0.5, a false discovery rate <0.01 identified 156 potential perichondrium markers (Table S1). Using a stricter criterion with an algorithm score of <0.25 and a fold‐change of more than 10‐fold (perichondrium versus each growth plate zone) identified 59 potential perichondrium markers that were highly expressed in perichondrium compared with growth plate cartilage (Table 2; Figs. 1 *A* and S1*A*). The top‐ranking marker for perichondrium, tenascin N (*Tnn*), showed mRNA levels in the perichondrium that were more than 150‐fold higher than in the growth plate, followed by asporin (*Aspn*; 161‐fold), tenascin C (*Tnc*; 68‐fold), and *Postn* (46‐fold; Table S1). Other candidate markers included transmembrane protein 119 (*Tmm119*; 50‐fold), *Lgals1* (33‐fold), *Robo2* (42‐fold), *Ptprz1* (32‐fold), cadherin 11 (*Cdh11;* 39‐fold), *Cdh2* (29‐fold), insulin‐like growth‐factor binding protein 7 (*Igfbp7*; 27‐fold), and *Thbs4* (18‐fold; Table S1). Our results included perichondrium markers that have been found to be specific for individual layers of the perichondrium by Bandyopadhyay and colleagues,^24^ including cambium markers *Dkk3* and *Lgals1*, as well as fibrous‐layer markers, cellular retinoic acid binding protein 1 (*Crabp1*) and tenomodulin (*Tnmd*)^24^ (Fig. 1 *B*). For all genes studied by both microarray and real‐time PCR, gene expression correlated very well between the two different techniques, supporting the validity of this approach (Fig. 1 *C*). Bioinformatics analysis of the 59 top‐ranked perichondrium markers identified significantly enriched molecular functions including PDGF‐ and IGF‐binding, biological processes including regulation of cell growth, cell adhesion, and response to hypoxia (Fig. S1*B*). Significantly enriched pathways included mineralization of bone, proliferation of connective tissue cells, development of vasculature, and migration of endothelial cells—all important during skeletal development and growth (Fig. S1*C*).

**Table 2:** Differentially Expressed Genes in Perichondrium (*n *= 59) Compared With Epiphyseal Cartilage

Gene symbol	PC (raw signal)	GP (raw signal)	PC vs. GP (fold‐change)	PC marker algorithm
*Tnn*	3245	21	157	0,04
*Aspn*	2236	14	161	0,05
*Tnc*	5856	86	68	0,05
*Postn*	11466	249	46	0,07
*Tmem119*	4712	93	50	0,07
*Col4a1*	2899	64	45	0,09
*Htra1*	3017	84	36	0,1
*Lgals1*	4186	125	34	0,1
*Cdh11*	1937	49	40	0,11
*Serpinf1*	5375	177	30	0,11
*Crabp1*	1808	51	35	0,12
*Col3a1*	11077	438	25	0,12
*Igfbp7*	2993	108	28	0,13
*Robo2*	1013	24	42	0,13
*S1pr1*	1174	32	36	0,14
*Cav1*	1281	38	34	0,14
*Vcan*	1023	26	39	0,14
*Fam167a*	1904	69	28	0,14
*Dmp1*	2395	98	25	0,15
*Ptprz1*	1069	33	33	0,15
*Csrp2*	1277	43	30	0,15
*Ptprd*	1983	79	25	0,15
*Tpm2*	2033	86	24	0,16
*Mamdc2*	1144	42	27	0,16
*Crip1*	2156	98	22	0,17
*Rgs5*	977	36	27	0,18
*Kazald1*	1088	43	25	0,18
*Ano6*	2385	121	20	0,18
*Mmp2*	5920	339	17	0,18
*Aqp1*	1094	46	24	0,18
*Kdr*	942	38	24	0,19
*Bglap*	734	26	28	0,19
*Col5a2*	7576	476	16	0,2
*S1pr3*	1918	110	18	0,2
*Cfh*	1766	101	18	0,21
*Cdh2*	593	20	30	0,21
*Igfbp3*	806	36	22	0,21
*Cgnl1*	888	43	21	0,21
*Satb2*	842	40	21	0,22
*Igf1*	1727	106	16	0,22
*Loxl1*	2393	158	15	0,22
*Lamb1*	809	40	20	0,23
*Maf*	960	52	19	0,23
*Epha3*	522	19	28	0,23
*Plvap*	1301	78	17	0,23
*Sepp1*	1751	116	15	0,23
*Col24a1*	2092	144	15	0,24
*Tm4sf1*	617	28	22	0,24
*Igsf10*	1593	107	15	0,24
*Thbs4*	760	40	19	0,24
*Col1a1*	12596	1001	13	0,24
*Lmo7*	1032	63	16	0,24
*Aplnr*	491	20	25	0,25
*Ifitm3*	1354	92	15	0,25
*Tgfb3*	1294	88	15	0,25
*Ptn*	1791	130	14	0,25
*Tagln2*	3380	264	13	0,25
*Emcn*	800	47	17	0,25
*Tnmd*	574	28	21	0,25

All genes have a one‐way ANOVA false discovery rate <0.01 and an algorithm marker <0.25. PC = perichondrium; GP = growth plate.

### Validation of epiphyseal cartilage dissection

**Figure 2:**
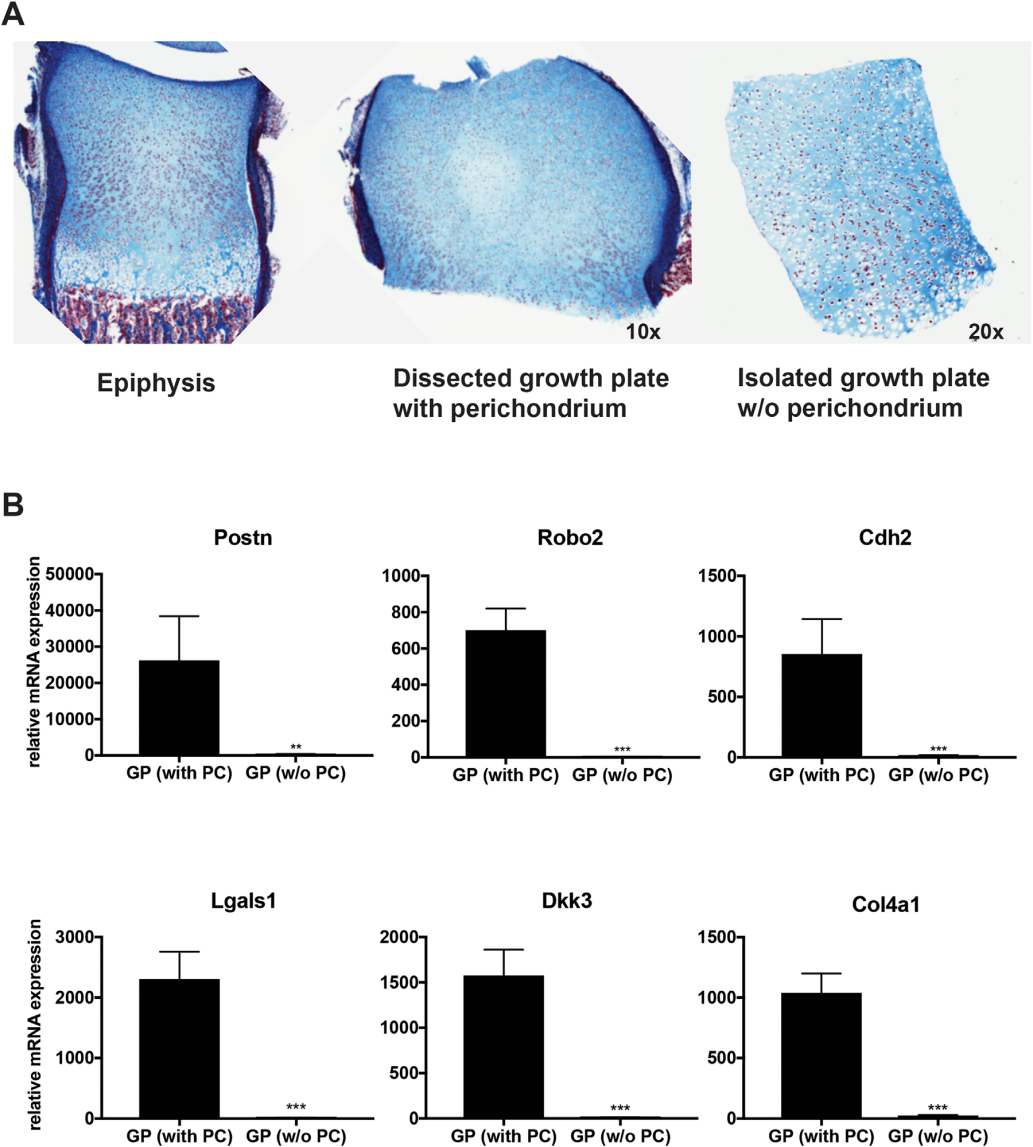
Experimental approach to study perichondrium origin. (*A*) Histological evaluation of isolated epiphyseal cartilage (GP) after trypan‐blue‐enhanced visualization of remaining perichondrium. Masson's trichrome staining of dissected cartilage pieces before and after (without perichondrium) complete removal of the perichondrium. (*B*) Quantitative real‐time PCR expression data of perichondrium selected markers in dissected epiphyseal growth plates with or without (w/o) perichondrium cells. GP = growth plate; PC = perichondrium; Postn = periostin; Robo2 = roundabout 2; Cdh2 = cadherin 2; Lgals1 = l‐galectin; Dkk3 = Dickkopf 3, Col4a1= collagen type IV alpha 1. **p < 0.01; ***p < 0.001.

After dissection of epiphyses from 3‐day‐old rats, the perichondrium was stained with trypan blue and then completely removed by dissection. To confirm the completeness of this dissection, one of every four dissected cartilage pieces was subjected to serial sectioning and histological analysis. No remaining perichondrium was detected on any of the sections from any of the pieces as assessed by two independent assessors. To further validate the epiphyseal dissection, perichondrium markers were confirmed using real‐time PCR comparing epiphyseal cartilage with some remaining perichondrium to the cartilage pieces dissected free of perichondrium (Fig. 2 *A*). As expected, expression levels of perichondrium markers were not detected or were minimal in the perichondrium‐free cartilage compared with the levels detected in pieces with some remaining perichondrium: *Postn* (81‐fold; *p* = 0.01), *Robo2* (301‐fold; *p *< 0.001), *Cdh2* (21‐fold; *p *< 0.001), *Dkk3* (105‐fold; *p *< 0.001), *Lgals1* (168‐fold; *p *< 0.001), and *Col4a1* (21‐fold; *p *< 0.001; Fig. 2 *B*).

### The outermost layer of chondrocyte pellets differentiate into a perichondrium‐like layer positive for cambium markers

**Figure 3:**
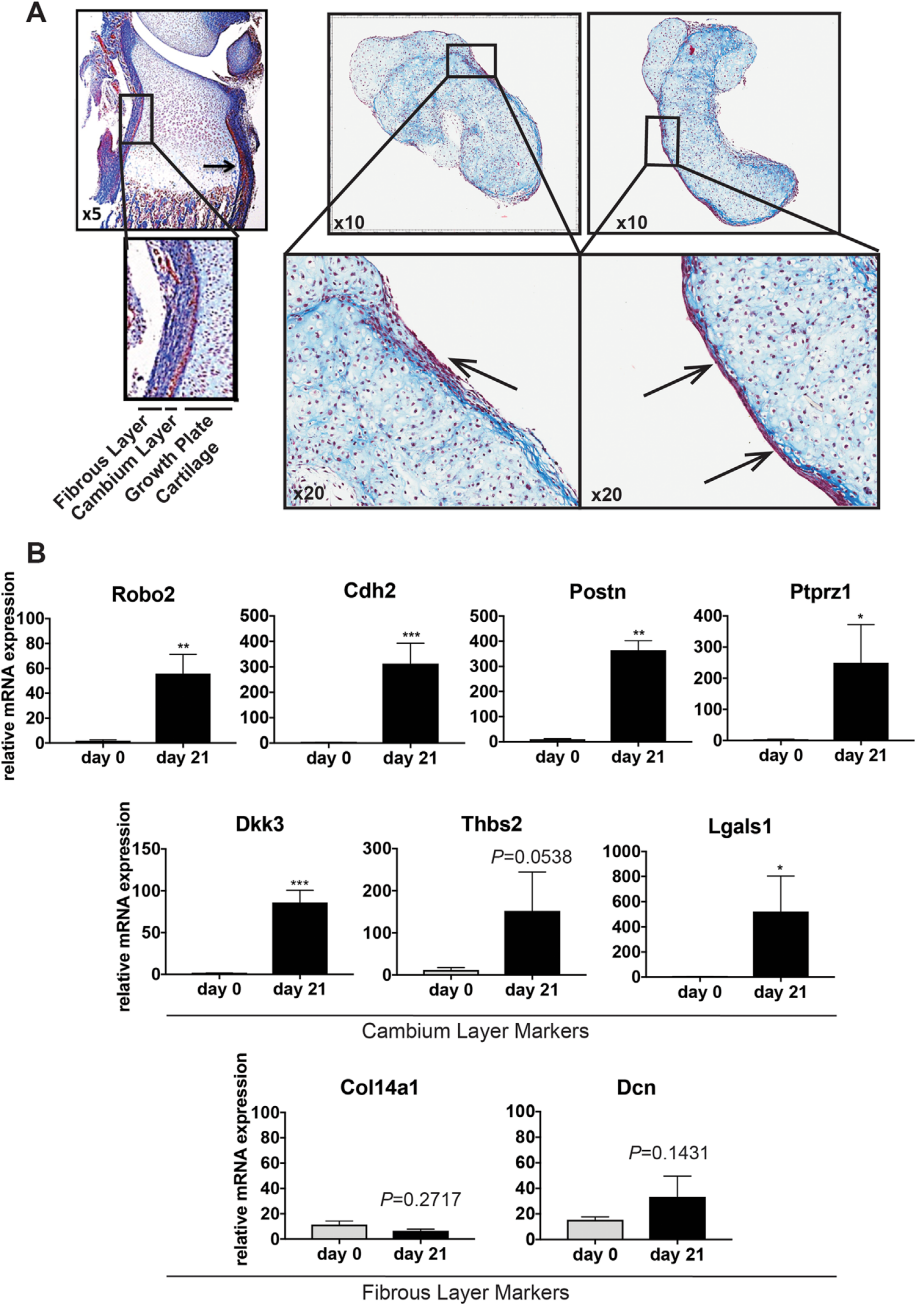
Perichondrium layer surrounding chondrocyte pellet cultures. (*A*) Masson's trichrome staining of rat proximal tibia epiphysis showing the different perichondrium layers (cambium and fibrous layer) and location of the growth plate cartilage, as well as two representative chondrocyte pellets after 21 days of culture. (*B*) Quantitative real‐time PCR expression data of novel perichondrium markers, as well as cambium and fibrous layer‐specific markers on primary extracted (d0), perichondrium‐free chondrocytes, or chondrocyte pellets cultured for 21 days (d21). Postn = periostin; Robo2 = roundabout 2; Cdh2 = cadherin 2; Col14a1= collagen type XIV alpha 1; Ptprz1 = protein tyrosine phosphatase receptor type z1; Lgals1 = l‐galectin; Dkk3 = Dickkopf 3; Thbs4 = thrombospondin 4; Dcn = decorin. *p < 0.05; **p < 0.01; ***p < 0.001.

To explore the hypothesis that epiphyseal chondrocytes have the ability to form the exterior perichondrium‐like layer that appears around chondrocyte pellets, we cultured chondrocytes from epiphyseal cartilage dissected free of surrounding perichondrium in pellets and studied the development of a perichondrium layer by histology and expression of perichondrium markers. Cultured chondrocyte pellets developed an exterior perichondrium‐like layer that was stained mostly red by Masson's trichrome, characteristic of dense connective tissue, thus similar to the cambium layer of the perichondrium (Fig. 3 *A*). As expected, at day 0 the mRNA levels of perichondrium markers were not detected or were very low. However, with time, the mRNA levels of perichondrium markers, *Robo2* (27‐fold; *p *< 0.01)*, Cdh2* (140‐fold; *p *< 0.001)*, Postn* (34‐fold; *p *< 0.01), and *Ptprz1* (80‐fold; *p *< 0.05), increased dramatically. Interestingly, molecular markers specific for the cambium layer, *Dkk3* (*p *< 0.001)*, Thbs2* (*p* = 0.054), and *Lgals1* (*p* = 0.0258), were upregulated, whereas fibrous‐layer marker expression *Col14a1* (*p* = 0.27) and decorin (*p* = 0.14) remained low after 21 days of culture (Fig. 3 *B*), despite the formation of a histologically distinct perichondrium‐like layer (Fig. 3).

**Figure 4:**
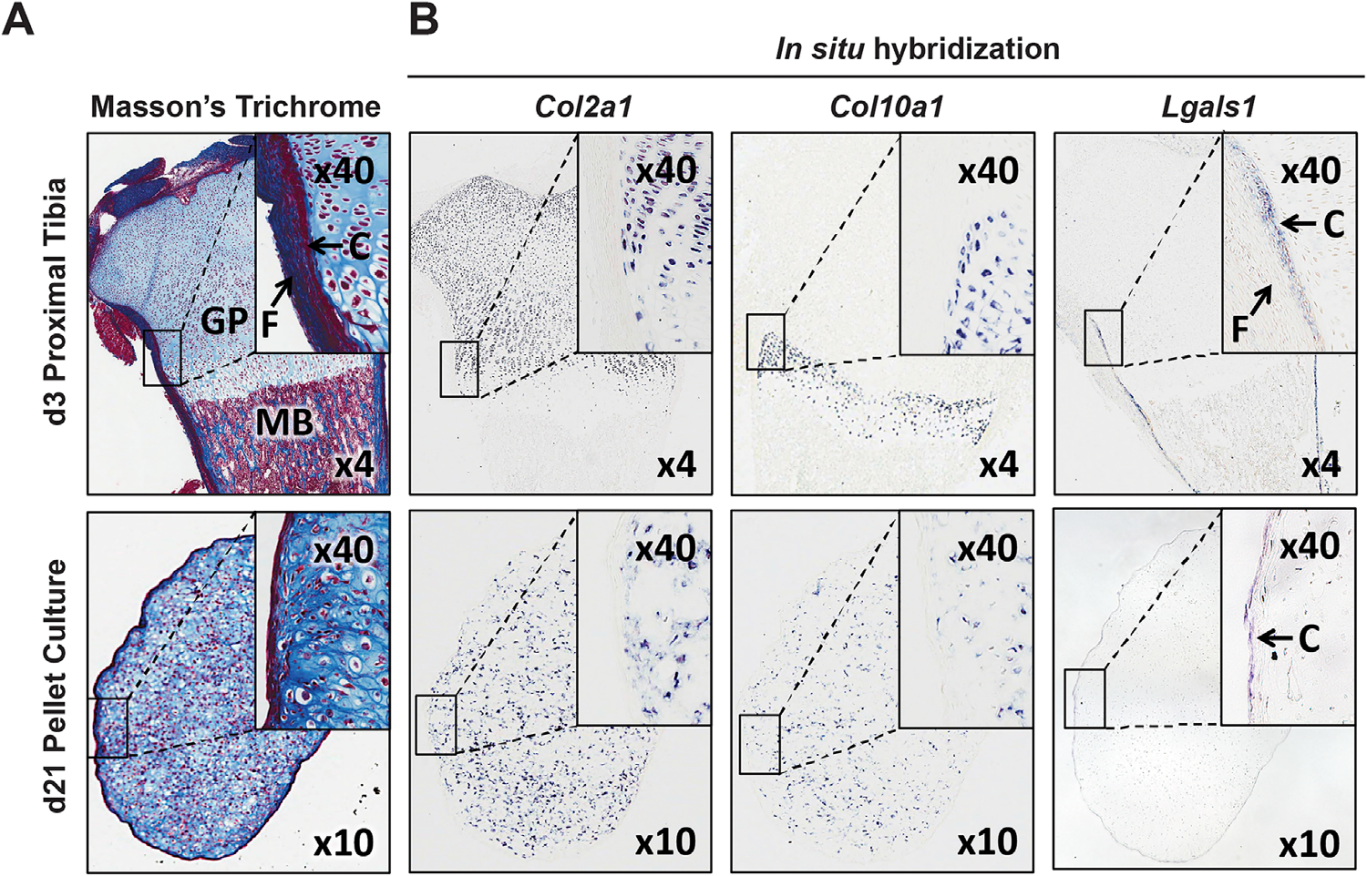
Localization of chondrocyte and perichondrium marker expression by in situ hybridization. (*A*) Masson's trichrome staining of day 3 postnatal rat proximal tibia including perichondrium layers (cambium (C) and fibrous layer (F)), growth plate cartilage (GP), and metaphyseal bone (MB), as well as representative chondrocyte pellets after 21 days of culture. (*B*) mRNA expression of chondrogenic (*Col2a1* and *Col10a1*) markers and a cambium‐specific perichondrium marker (*Lgals1*) in postnatal day 3 (d3) rat proximal tibia and chondrocyte pellet cultures on day 21(d21). The magnification of individual pictures taken is mentioned, with the viewing fields for rat proximal tibia or chondrocyte pellet sections kept the same for histology and in situ hybridization examination. GP = growth plate cartilage; MB = metaphyseal bone; C = cambium (inner) layer; F = fibrous (outer) layer of perichondrium; *Col2a1* = collagen type II alpha 1; *Col10a1* = collagen type X alpha 1; *Lgals1* = l‐galectin.

To confirm that the upregulated perichondrium markers were located in the perichondrium‐like layer only, expression of perichondrium marker *Lgals1* chondrocyte markers *Col2a1* and *ColX* were studied by in situ hybridization. *Col2a1* and *ColX* were detected in all cells of the pellet except in the cells of the perichondrium‐like layer (Figs. 4A, B). In contrast, *Lgals1* mRNA expression was only detected in the outermost cells of the perichondrium‐like layer (Figs. 3 and 4).

## Discussion

Mammalian chondrocytes cultured as high‐density cell pellets develop a surrounding perichondrium‐like layer.^13^ To explore the cellular origin of this cell layer, we identified perichondrium‐specific markers and developed a dissection technique that enabled complete removal of all surrounding perichondrium from epiphyseal cartilage collected from 3‐day‐old rats. Despite the removal of contaminating perichondrium, a perichondrial‐like layer surrounding the chondrocyte pellets developed. This histological observation was also supported by dramatic increases in mRNA levels of perichondrium markers. Interestingly, cambium, but not fibrous‐layer markers, was upregulated in the perichondrium‐like layer, suggesting that postnatal epiphyseal chondrocytes have the ability to differentiate into perichondrium cells of the cambium layer. Our findings thus confirm and extend previous studies suggesting that chondrocytes can differentiate into perichondrium cells.^11^, ^12^, ^13^, ^14^, ^15^, ^16^

To identify perichondrium markers, an algorithm that gave high rank to genes that were highly expressed in the perichondrium compared with each of the major zones of the growth plate (resting, proliferative, and hypertrophic) was used; it identified 59 genes that were highly expressed in perichondrium compared with growth plate cartilage (Tables 2 and S1). The top‐ranked markers included extracellular matrix (ECM) proteins *Tnn*, *Aspn*, and *Tnc*, important for cartilage ECM organization and mineralization and also as modifiers of TGF‐beta1 and Wnt/β‐catenin signaling^25^, ^26^ during chondrogenesis,^27^, ^28^ indicating that several of the identified markers also may be biologically important in the developing skeleton. Interestingly, genes involved in ECM structure and assembly such as *Aspn*, versican, *Tnn*, and *Tnc*, and collagens such as Col4a1 were among the most highly expressed genes in perichondrium compared with growth plate cartilage, underscoring the extensive modeling of ECM that occurs in the perichondrium during growth. Moreover, Ingenuity Pathway Analysis (QIAGEN Bioinformatics) showed that many genes involved in bone mineralization and vasculature development were upregulated in the perichondrium. Furthermore, gene ontology analysis revealed that PDGF‐binding proteins such as COL1A1, COL3A1, and COL4A1, were significantly enriched in perichondrium. Interestingly, IGF‐1 and IGF‐binding proteins, such as IGFBP3 and IGFBP7, were also enriched in perichondrium. This indicates that the perichondrium may be important in modulating the local effects of growth hormone and IGFs at the growth plate. Though a detailed functional investigation is needed, our data indicate some potential candidates involved in the crucial processes of bone development and growth.

We found only three published studies that presented a wide range of molecular markers for perichondrium by utilizing microarrays.^24^, ^29^, ^30^ Bandyopadhyay and colleagues^24^ looked for novel molecular markers by comparing the perichondrium against the periosteum of embryonic chicken. The other two studies used a similar approach, but did not include all the zones of growth plate; therefore, they are not directly comparable to our study. Our findings identified several novel markers, including several genes previously shown to be highly expressed in the perichondrium cambium layer, such as *Lgals1*, *Dkk3*, fibrous *Crabp1*, and *Tnmd*,^24^, ^29^, ^30^ thus confirming the validity of our approach. Interestingly, musculoaponeurotic fibrosarcoma oncogene homolog (*Maf*) and *Thbs4*, which belong to the same family as the cambium‐specific markers *Mafb* and *Thbs2*, respectively,^24^ were also detected as perichondrium markers in our study. Another top‐ranked perichondrium marker, *Postn* encodes a secreted ECM protein essential for postnatal growth development.^31^ In this study, we have detected physiological markers highly expressed in the perichondrium. However, functional analyses are still needed to elucidate the exact roles of these perichondrium genes in the process of skeletogenesis.

We reasoned that complete removal of contaminating perichondrium is critical to the interpretation of the results because a new perichondrium might form, even from a relatively small number of contaminating perichondrial cells. Hence we developed a dissection technique that allowed for consistent and complete removal of all perichondrium from the cartilage pieces used for extraction of chondrocytes. As mentioned above, our findings are consistent with other studies suggesting that chondrocytes may differentiate into perichondrial cells.^11^, ^12^, ^13^, ^14^, ^15^ These previous studies also attempted to completely remove contaminating perichondrium, but did not present a strategy beyond careful dissection to confirm the complete stripping of perichondrium.^11^, ^12^, ^13^ In addition, we found that the cells that produced the perichondrium‐like layer express cambium, but not fibrous‐layer markers. Our study thus confirms and extends the previous studies, which have reported a perichondrium‐like layer forming around cartilage and chondrocyte pellets after 10 to 21 days of culture.^11^ Interestingly, Ballock and Reddi^13^ reported that a perichondrium‐like layer forms in a dose‐dependent matter when the concentration of FBS is 1% or higher, but not at FBS concentrations of 0.1% or lower. This finding, taken together with our finding that it is chondrocytes that form the perichondrium, suggests that FBS contains one or several factors that promote perichondrial differentiation of epiphyseal chondrocytes at the surface of chondrocyte pellet cultures. Our findings are consistent with the findings of Hyde and colleagues who used collagen type‐II lineage‐tracing and showed that early collagen type II‐expressing cells may differentiate into perichondrium.^1^ Our findings are also largely consistent with the findings that postnatal chondrocytes, and even postnatal hypertrophic chondrocytes, have retained pluripotent properties and can differentiate into other skeletal cells, thus countering the dogma that postnatal growth plate chondrocytes are terminally differentiated cells.^5^, ^17^

In conclusion, this study provides evidence that postnatal chondrocytes can differentiate into perichondrial cells of the cambium layer. In addition, the novel perichondrium markers we identified may be useful tools in future studies of skeletal development, especially in relation to the cellular interactions of cartilage and perichondrium. Further studies are needed to explore the molecular signals that induce the transdifferentiation of chondrocytes into perichondrial and other skeletal cells during bone development.

## Disclosures

ON has received speakers’ honoraria from Pfizer and Lilly, and research support from Novo Nordisk. All authors state that they have no conflicts of interest.

## Supporting information

Supporting Data S1.
