# Supplementary material for: Evidence That Rat Chondrocytes Can Differentiate Into Perichondrial Cells
Source: JBMR Plus. 2018 Jun 7;2(6):351–61. doi: 10.1002/jbm4.10056 (PMC6237212; doi:10.1002/jbm4.10056)
Supplement: Supplementary file 1 — Supporting Data S1. [file JBM4-2-351-s001.docx]

**Title: Ev**

**Supplemental Data:**

**Supplementary Figure 1.** Genes upregulated in PC are involved in processes important for skeletal development. A) Heat map of the 59 genes upregulated in PC compared to epiphyseal cartilage (defined by false discovery rate < 0.01 Perichondrium vs. Growth Plate). Color scale represents log transformed intensity values of microarray data. B) Gene ontology analysis of the 59 genes upregulated in PC. Only significant GO terms are shown (Benjamini *P* < 0.05). C) IPA analysis of the 59 genes revealed involvement in Mineralization of bone, Proliferation of connective tissue cells, Development of vasculature and Migration of endothelial cells. Color scale indicates the degree of upregulation.


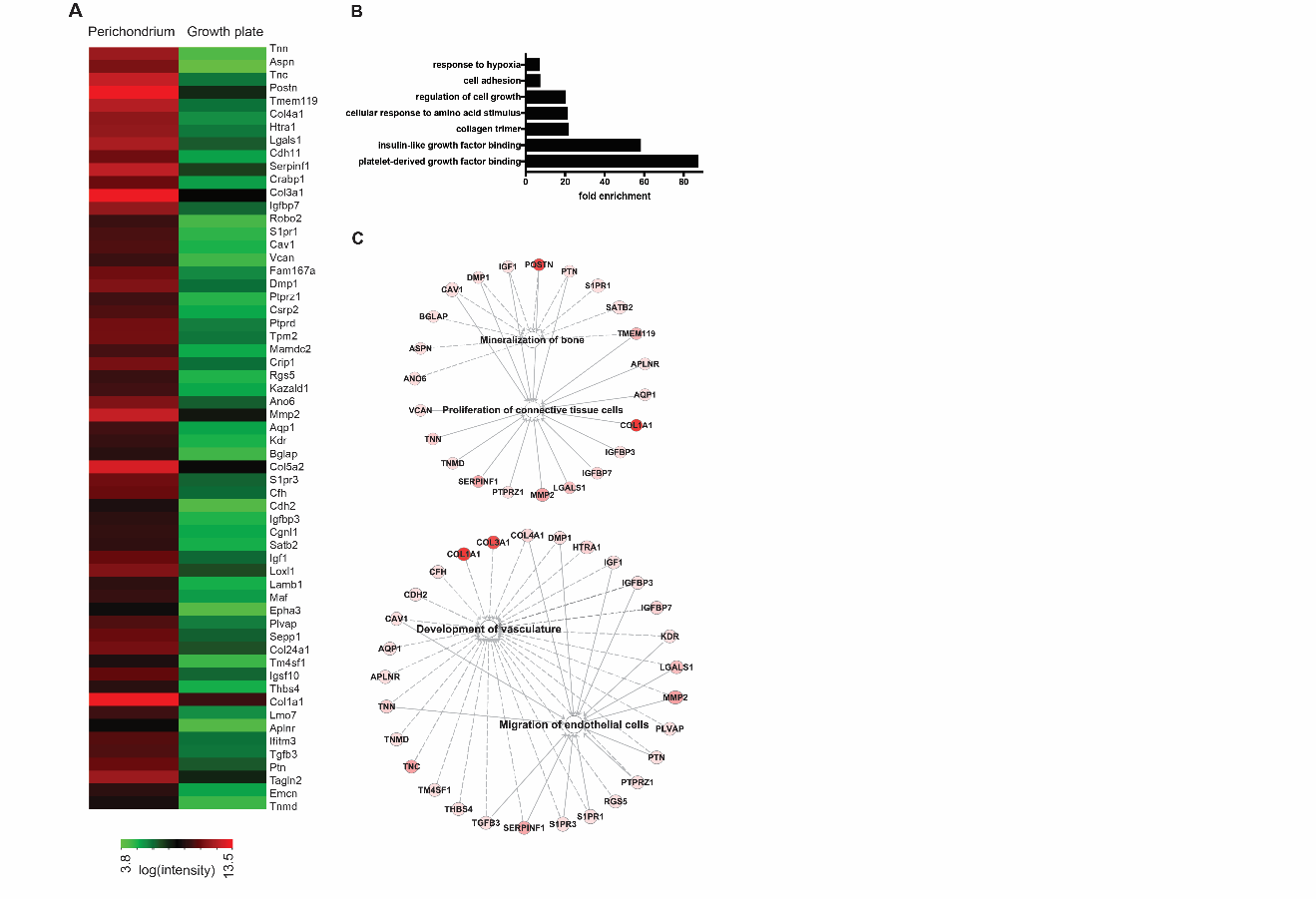


**Table S1.** Differentially expressed genes in perichondrium (n=156) compared to epiphyseal cartilage. All genes have a one-way ANOVA false discovery rate < 0.01 and an algorithm marker < 0.5.

**Table S1**

| Gene Symbol | PC  (raw signal) | GP  (raw signal) | PC vs GP  (Fold-change) | PC marker  algorithm |
| --- | --- | --- | --- | --- |
| Tnn | 3245 | 21 | 157 | 0,04 |
| Aspn | 2236 | 14 | 161 | 0,05 |
| Tnc | 5856 | 86 | 68 | 0,05 |
| Postn | 11466 | 249 | 46 | 0,07 |
| Tmem119 | 4712 | 93 | 50 | 0,07 |
| Col4a1 | 2899 | 64 | 45 | 0,09 |
| Htra1 | 3017 | 84 | 36 | 0,1 |
| Lgals1 | 4186 | 125 | 34 | 0,1 |
| Cdh11 | 1937 | 49 | 40 | 0,11 |
| Serpinf1 | 5375 | 177 | 30 | 0,11 |
| Crabp1 | 1808 | 51 | 35 | 0,12 |
| Col3a1 | 11077 | 438 | 25 | 0,12 |
| Igfbp7 | 2993 | 108 | 28 | 0,13 |
| Robo2 | 1013 | 24 | 42 | 0,13 |
| S1pr1 | 1174 | 32 | 36 | 0,14 |
| Cav1 | 1281 | 38 | 34 | 0,14 |
| Vcan | 1023 | 26 | 39 | 0,14 |
| Fam167a | 1904 | 69 | 28 | 0,14 |
| Dmp1 | 2395 | 98 | 25 | 0,15 |
| Ptprz1 | 1069 | 33 | 33 | 0,15 |
| Csrp2 | 1277 | 43 | 30 | 0,15 |
| Ptprd | 1983 | 79 | 25 | 0,15 |
| Tpm2 | 2033 | 86 | 24 | 0,16 |
| Mamdc2 | 1144 | 42 | 27 | 0,16 |
| Crip1 | 2156 | 98 | 22 | 0,17 |
| Rgs5 | 977 | 36 | 27 | 0,18 |
| Kazald1 | 1088 | 43 | 25 | 0,18 |
| Ano6 | 2385 | 121 | 20 | 0,18 |
| Mmp2 | 5920 | 339 | 17 | 0,18 |
| Aqp1 | 1094 | 46 | 24 | 0,18 |
| Kdr | 942 | 38 | 24 | 0,19 |
| Bglap | 734 | 26 | 28 | 0,19 |
| Col5a2 | 7576 | 476 | 16 | 0,2 |
| S1pr3 | 1918 | 110 | 18 | 0,2 |
| Cfh | 1766 | 101 | 18 | 0,21 |
| Cdh2 | 593 | 20 | 30 | 0,21 |
| Igfbp3 | 806 | 36 | 22 | 0,21 |
| Cgnl1 | 888 | 43 | 21 | 0,21 |
| Satb2 | 842 | 40 | 21 | 0,22 |
| Igf1 | 1727 | 106 | 16 | 0,22 |
| Loxl1 | 2393 | 158 | 15 | 0,22 |
| Lamb1 | 809 | 40 | 20 | 0,23 |
| Maf | 960 | 52 | 19 | 0,23 |
| Epha3 | 522 | 19 | 28 | 0,23 |
| Plvap | 1301 | 78 | 17 | 0,23 |
| Sepp1 | 1751 | 116 | 15 | 0,23 |
| Col24a1 | 2092 | 144 | 15 | 0,24 |
| Tm4sf1 | 617 | 28 | 22 | 0,24 |
| Igsf10 | 1593 | 107 | 15 | 0,24 |
| Thbs4 | 760 | 40 | 19 | 0,24 |
| Col1a1 | 12596 | 1001 | 13 | 0,24 |
| Lmo7 | 1032 | 63 | 16 | 0,24 |
| Aplnr | 491 | 20 | 25 | 0,25 |
| Ifitm3 | 1354 | 92 | 15 | 0,25 |
| Tgfb3 | 1294 | 88 | 15 | 0,25 |
| Ptn | 1791 | 130 | 14 | 0,25 |
| Tagln2 | 3380 | 264 | 13 | 0,25 |
| Emcn | 800 | 47 | 17 | 0,25 |
| Tnmd | 574 | 28 | 21 | 0,25 |
| Sema3b | 477 | 20 | 24 | 0,26 |
| Lox | 5769 | 481 | 12 | 0,26 |
| Evi2a | 438 | 17 | 25 | 0,26 |
| Mylk | 1711 | 132 | 13 | 0,27 |
| Lhfp | 804 | 52 | 16 | 0,27 |
| Fbn2 | 585 | 33 | 18 | 0,28 |
| Olfml2b | 824 | 56 | 15 | 0,28 |
| Myo1b | 686 | 46 | 15 | 0,29 |
| Mfap4 | 406 | 19 | 22 | 0,29 |
| Prss23 | 386 | 17 | 23 | 0,29 |
| Spp1 | 2529 | 225 | 11 | 0,29 |
| Ppm1l | 458 | 24 | 19 | 0,29 |
| Tcf7 | 703 | 49 | 14 | 0,3 |
| Twist1 | 590 | 39 | 15 | 0,3 |
| Filip1l | 404 | 20 | 20 | 0,3 |
| Mfap5 | 800 | 62 | 13 | 0,31 |
| Col12a1 | 775 | 60 | 13 | 0,31 |
| Col8a1 | 496 | 31 | 16 | 0,31 |
| Akap2 | 1168 | 102 | 11 | 0,32 |
| Etl4 | 437 | 25 | 17 | 0,32 |
| Snx7 | 606 | 44 | 14 | 0,32 |
| Pdpn | 1512 | 140 | 11 | 0,32 |
| Prrx2 | 569 | 40 | 14 | 0,32 |
| Cd248 | 799 | 66 | 12 | 0,33 |
| Folr1 | 513 | 36 | 14 | 0,33 |
| Egr1 | 1170 | 108 | 11 | 0,33 |
| Crip2 | 1292 | 124 | 10 | 0,34 |
| Csrp1 | 1976 | 203 | 10 | 0,34 |
| Phex | 299 | 13 | 23 | 0,34 |
| Col4a2 | 1403 | 139 | 10 | 0,34 |
| Entpd3 | 470 | 33 | 14 | 0,35 |
| Meox2 | 289 | 13 | 23 | 0,35 |
| Timp1 | 1520 | 160 | 9 | 0,36 |
| Folr2 | 490 | 38 | 13 | 0,36 |
| Cd93 | 386 | 26 | 15 | 0,37 |
| Lmna | 971 | 98 | 10 | 0,37 |
| Cd36 | 760 | 72 | 11 | 0,37 |
| Slc40a1 | 2239 | 257 | 9 | 0,37 |
| Endod1 | 593 | 53 | 11 | 0,38 |
| Sema6d | 452 | 36 | 13 | 0,38 |
| Svil | 739 | 72 | 10 | 0,38 |
| Fam198b | 666 | 63 | 11 | 0,38 |
| Fam171a1 | 844 | 87 | 10 | 0,38 |
| Ptgfrn | 855 | 89 | 10 | 0,39 |
| Stk17b | 725 | 73 | 10 | 0,39 |
| Hs3st6 | 394 | 31 | 13 | 0,39 |
| B2m | 870 | 94 | 9 | 0,4 |
| Marcks | 4548 | 580 | 8 | 0,4 |
| Arhgap24 | 805 | 86 | 9 | 0,4 |
| Rai14 | 916 | 103 | 9 | 0,41 |
| Wisp1 | 656 | 68 | 10 | 0,41 |
| Ptprb | 283 | 17 | 16 | 0,41 |
| Sparcl1 | 407 | 35 | 12 | 0,41 |
| Cd1d1 | 344 | 26 | 13 | 0,41 |
| Zeb2 | 239 | 12 | 19 | 0,42 |
| Mxra8 | 1693 | 216 | 8 | 0,42 |
| Reep3 | 944 | 112 | 8 | 0,42 |
| Mrc1 | 278 | 18 | 15 | 0,43 |
| Six1 | 264 | 17 | 16 | 0,43 |
| Epb41l3 | 318 | 25 | 13 | 0,43 |
| S100a4 | 4589 | 667 | 7 | 0,45 |
| Trim2 | 455 | 48 | 10 | 0,45 |
| Dkk3 | 574 | 66 | 9 | 0,45 |
| Slit2 | 249 | 17 | 15 | 0,45 |
| Ccdc3 | 365 | 34 | 11 | 0,45 |
| Arhgap28 | 377 | 36 | 10 | 0,46 |
| Tspan2 | 795 | 101 | 8 | 0,46 |
| Kcnk6 | 1048 | 141 | 7 | 0,46 |
| Scara3 | 700 | 87 | 8 | 0,46 |
| Ctsc | 563 | 66 | 9 | 0,46 |
| Dclk1 | 880 | 116 | 8 | 0,47 |
| Lurap1l | 413 | 43 | 10 | 0,47 |
| Lrrc17 | 1305 | 183 | 7 | 0,47 |
| Hck | 336 | 32 | 11 | 0,47 |
| Rbms3 | 363 | 36 | 10 | 0,47 |
| Vstm4 | 433 | 47 | 9 | 0,47 |
| Mid1 | 463 | 52 | 9 | 0,47 |
| Adamts4 | 290 | 25 | 12 | 0,48 |
| Prmt8 | 238 | 17 | 14 | 0,48 |
| Mest | 2729 | 412 | 7 | 0,48 |
| Mgst3 | 574 | 70 | 8 | 0,48 |
| Col18a1 | 454 | 51 | 9 | 0,48 |
| Cpq | 942 | 130 | 7 | 0,48 |
| Foxp1 | 820 | 111 | 7 | 0,48 |
| Tppp3 | 582 | 73 | 8 | 0,48 |
| Gpnmb | 898 | 124 | 7 | 0,48 |
| Itga11 | 447 | 51 | 9 | 0,48 |
| Nr2f2 | 217 | 14 | 15 | 0,48 |
| St6gal1 | 468 | 55 | 8 | 0,49 |
| Slc6a15 | 304 | 29 | 11 | 0,49 |
| Ltbp4 | 402 | 45 | 9 | 0,49 |
| Mmp23 | 670 | 89 | 8 | 0,49 |
| Dysf | 731 | 99 | 7 | 0,49 |
| Dpt | 286 | 26 | 11 | 0,49 |
| Abracl | 504 | 62 | 8 | 0,5 |
| Coro1c | 2528 | 397 | 6 | 0,5 |
| Tfpi | 427 | 50 | 9 | 0,5 |
